# Activation of Anthracene Endoperoxides in *Leishmania* and Impairment of Mitochondrial Functions

**DOI:** 10.3390/molecules23071680

**Published:** 2018-07-10

**Authors:** Gerald Geroldinger, Matthias Tonner, Werner Fudickar, Sritama De Sarkar, Aishwarya Dighal, Lianet Monzote, Katrin Staniek, Torsten Linker, Mitali Chatterjee, Lars Gille

**Affiliations:** 1 Institute of Pharmacology and Toxicology, Department of Biomedical Sciences, University of Veterinary Medicine, A-1210 Vienna, Austria; Gerald.Geroldinger@vetmeduni.ac.at (G.G.); Matthias-Stefan.Tonner@vetmeduni.ac.at (M.T.); Katrin.Staniek@vetmeduni.ac.at (K.S.); 2 Department of Organic Chemistry, Institute of Chemistry, University of Potsdam, D-14476 Golm/Potsdam, Germany; wfudicka@uni-potsdam.de (W.F.); linker@uni-potsdam.de (T.L.); 3 Department of Pharmacology, Institute of Postgraduate Medical Education & Research, Kolkata 700020, India; sd0900@gmail.com (S.D.S.); aishwarya.dighal@gmail.com (A.D.); ilatimc@gmail.com (M.C.); 4 Parasitology Department, Institute of Tropical Medicine “Pedro Kouri”, Havana 10400, Cuba; monzote@ipk.sld.cu

**Keywords:** Leishmania, endoperoxides, EPR spectroscopy, mitochondria, radicals

## Abstract

Leishmaniasis is a vector-borne disease caused by protozoal *Leishmania*. Because of resistance development against current drugs, new antileishmanial compounds are urgently needed. Endoperoxides (EPs) are successfully used in malaria therapy, and experimental evidence of their potential against leishmaniasis exists. Anthracene endoperoxides (AcEPs) have so far been only technically used and not explored for their leishmanicidal potential. This study verified the in vitro efficiency and mechanism of AcEPs against both *Leishmania* promastigotes and axenic amastigotes (*L. tarentolae* and *L. donovani*) as well as their toxicity in J774 macrophages. Additionally, the kinetics and radical products of AcEPs’ reaction with iron, the formation of radicals by AcEPs in *Leishmania*, as well as the resulting impairment of parasite mitochondrial functions were studied. Using electron paramagnetic resonance combined with spin trapping, photometry, and fluorescence-based oximetry, AcEPs were demonstrated to (i) show antileishmanial activity in vitro at IC_50_ values in a low micromolar range, (ii) exhibit host cell toxicity in J774 macrophages, (iii) react rapidly with iron (II) resulting in the formation of oxygen- and carbon-centered radicals, (iv) produce carbon-centered radicals which could secondarily trigger superoxide radical formation in *Leishmania*, and (v) impair mitochondrial functions in *Leishmania* during parasite killing. Overall, the data of different AcEPs demonstrate that their structures besides the peroxo bridge strongly influence their activity and mechanism of their antileishmanial action.

## 1. Introduction

Leishmaniasis is a protozoal disease which is endemic especially in tropical and subtropical regions [1,2]. In spite of the well-established use of chemotherapeutics and multiple therapy options, the disease presents a growing threat to millions of people [3]. Depending on the particular species of *Leishmania* and other factors, various clinical manifestations occur, ranging from self-resolving or disfiguring cutaneous lesions to life-threatening visceral forms affecting the liver and the spleen. The present antileishmanial drugs show increasing resistance, are expensive, and exhibit significant side effects [4], endorsing the need to identify new drug classes and targets [5,6]. In malaria, endoperoxides (EPs) are an integral part of chemotherapy [7]. The mechanism of action of antimalarial EPs is based on the fact that plasmodia reside inside host erythrocytes, and degrade hemoglobin to products which react with EPs [8].

*Leishmania* amastigotes persist inside macrophages which contain variable amounts of heme and other sources of iron. However, depending on the localization of macrophages in certain tissues, they can be involved in the phagocytosis of erythrocytes [9]. This leads to an accumulation of heme compounds in the phagolysosome of the leishmanial host cells, which can be acquired by *Leishmania* via the LHR1 transporter [10].

Previous studies have elucidated that EPs, such as ascaridole (Asc) and artemisinin (Art), are effective against *Leishmania* in vitro [11,12,13]. Mechanistic studies with these EPs [14,15,16] suggest that the reductive cleavage of the endoperoxide bridge in EPs upon reaction with Fe^2+^ causes the formation of cytotoxic radicals. It has been shown that the residual structure of these EPs strongly influenced their reactivity against different iron sources, pharmacokinetic availability and the ability to perform non-peroxide-related reactions.

Therefore, as a consequence, it was a logical step to explore the antileishmanial properties of EPs with structures different from Asc and Art. A class of new EPs was developed from the reaction of substituted anthracenes (Ac) with singlet oxygen. These anthracene endoperoxides (AcEPs) were initially designed for technical applications as oxygen carriers, photoswitches, or photoresists [17]. The antileishmanial potential of these compounds has not been studied so far. Ac and anthraquinone (AQ) have played a role in the basic structure of certain drugs [18]. The aim of the current study is to explore potential antileishmanial activities of these compounds in vitro using cell-free systems as well as *Leishmania tarentolae*, *Leishmania donovani* and a mouse macrophage cell line. The data obtained demonstrate that depending on the structure of the individual AcEPs, they exhibit potent antileishmanial activity. Therefore, this class of compounds may be a new starting point for the development of antileishmanials.

## 2. Materials and Methods

### 2.1.Chemicals

2,2,6,6-Tetramethylpiperidine (TEMP), 5,5-dimethyl-1-pyrroline N-oxide (DMPO), antimycin A, artemisinin (Art), brain heart infusion (BHI) medium, carbonyl cyanide m-chlorophenylhydrazone (CCCP), hemin, M199 medium, paraffin oil, penicillin-streptomycin solution, pentamidine (Pen) isethionate, resazurin, rose bengal (RB), and thiazolyl blue tetrazolium bromide (MTT) were purchased from Sigma-Aldrich (St. Louis, MO, USA). 3-(4,5-Dimethylthiazol-2-yl)-5-(3-carboxymethoxyphenyl)-2-(4-sulphophenyl)-2H tetrazolium (MTS), inner salt was purchased from Promega (Madison, WI, USA). Phenazine methosulfate (PMS) was purchased from SRL (Mohali, India). Butylated hydroxytoluene was from Roche (Basel, Switzerland). Dulbecco’s Modified Eagle Medium (DMEM) was from Thermo Fisher Scientific (Waltham, MA, USA) and low-endotoxin fetal calf serum (FCS) from Bio&Sell (Nürnberg, Germany). Fetal bovine serum (FBS) was purchased from Gibco (Carlsbad, CA, USA). Dimethyl sulfoxide (DMSO) and formaldehyde were from Roth (Karlsruhe, Germany). Diethylenetriaminepentaacetic acid (DTPA), acetonitrile, anthraquinone (AQ), FeSO_4_, glucose, HCl, H_2_SO_4_, KCl, K_2_HPO_4_, KH_2_PO_4_, methanol, NaCl, Na_2_HPO_4,_ sodium dithionite, and xylenol orange (XO) were obtained from Merck (Darmstadt, Germany). Oligomycin was obtained from EMD Chemicals (San Diego, CA, USA). Hydroxyl-3-methoxycarbonyl-2,2,5,5-tetramethylpyrrolidine hydrochloride (CMH) was obtained from Noxygen (Elzach, Baden-Württemberg, Germany). Anthracene (Ac) was obtained from Fluka AG (Buchs, Switzerland). Schneider’s Drosophila Medium was purchased from Biowest (Nuaillé, France). CO_2_ was purchased form Air Liquide (Schwechat, Austria). Ascaridole (Asc) was synthesized according to a previously published procedure [19]. Yeast extract powder was supplied by Amresco (Solon, OH, USA). Deferoxamine (DFO) was obtained from Novartis (Basel, Switzerland). Yeast extract medium (YEM) consisted of 20.7 g/L yeast extract powder, 1.2 g/L K_2_HPO_4_, 0.2 g/L KH_2_PO_4_, 2.9 g/L glucose, pH 7.4. Phosphate-buffered saline (PBS) contained 136 mM NaCl, 1.15 mM KH_2_PO_4_, 14 mM Na_2_HPO_4_, and 2.7 mM KCl, at pH 7.4. The anthracene endoperoxides (AcEP1117, AcEP1118, AcEP1129, and AcEP1130) were kindly provided by Dr. Werner Fudickar and Prof. Torsten Linker (Department of Organic Chemistry, Institute of Chemistry, University of Potsdam) [20,21]. The purity of the compounds was 99%.

### 2.2. Leishmania Tarentolae Promastigotes Culture

*Leishmania tarentolae* promastigotes (LtP) strain P10 from Jena Bioscience (Jena, Germany) were cultivated in brain heart infusion (BHI) medium (37 g/L) that was supplemented with 5 mg/L hemin, 25,000 U/L penicillin and 25 mg/L streptomycin at 26 °C. LtP were always used one day after being passaged, when the cells were in the logarithmic growth phase. The cell count was determined by measuring the optical density (OD) at a wavelength of 600 nm using a U1100 photometer (Hitachi, Tokyo, Japan). The number of the cells was subsequently calculated according to a calibration curve [22].

### 2.3. Axenic Leishmania Tarentolae Amastigotes Culture

An aliquot of the original LtP culture broth that was equivalent to 108 × 10^6^ cells was centrifuged at 2000× *g* for 10 min (Sorvall RC26 Plus, rotor: SH-3000, Thermo Fisher Scientific, Waltham, MA, USA). The resulting supernatant was discarded, and the cell pellet was resuspended in 10 mL of Schneider’s medium (adjusted to a pH of 4.5 using HCl; containing 50% FCS, 25,000 U/L penicillin, and 25 mg/L streptomycin). The cell suspension was transferred and cultured in a 50 mL TubeSpin bioreactor at 32 °C. Every 3–4 days, the cells were passaged. Therefore, the cell broth was centrifuged at 2000× *g* for 10 min, and the supernatant was discarded. The cells were resuspended in fresh medium. During the first 14 days, after the culture was started, the cells transformed from the promastigote to the axenic amastigote state (axLtA). Subsequent to this initial period, the axenic amastigotes were passaged every 3–4 days. To determine cell density of the broth, an aliquot of the suspension, usually 1 mL, was transferred into an Eppendorf tube. The cells were then diluted 1:2 up to 1:40 using PBS, depending on the cell density of the broth. An aliquot of 20 µL of this dilution was further diluted 1:2 with a 2% formaldehyde solution (in PBS). Of this final suspension, 10 µL were transferred to each counting chamber of a standard Neubauer chamber (VWR, Wien, Austria). The cells were incubated for 5 min at 25 °C to let them sediment. Photographs of the counting chambers were taken using a light microscope (Lacerta LIS-5 microscope, Lacerta GmbH, Vienna, Austria) with an attached camera. The photos were obtained at a magnification of 100×. Four photos of every counting chamber were taken, resulting in a total of eight photos. The pictures were then analyzed with the Fiji ImageJ (2.0.0) software (Wayne Rasband, National Institutes of Health (NIH), Bethesda, Maryland (MD), USA). The results were averaged giving the final cell number of the culture broth.

### 2.4. Leishmania Donovani Promastigotes Culture

*Leishmania donovani* (PS14) were originated from the splenic aspirate of a patient with visceral leishmaniasis. Typing of the species was done by a restriction fragment length polymorphism (RFLP) method. The parasites were cultivated in M199 medium supplemented with 20% FBS, penicillin G (50,000 IU/L), streptomycin (50 mg/L), and hemin (5 µM) at 24 °C for transformation. After 1–2 weeks, the successfully transformed parasites (*Leishmania donovani* promastigotes, LdP) were transferred to M199 medium supplemented with 10% FBS, penicillin G (50,000 IU/L), streptomycin (50 mg/L), and hemin (5 µM) in 12.5 cm^2^ flasks (HiMedia, Mumbai, India) at 24 °C. The cells were sub-cultured every 48–72 h, 1 × 10^6^ cells/mL being the inoculum.

### 2.5. Macrophage Culture

The murine macrophage cell line J774A.1 (mouse, ATCC, TIB-67™) was cultivated in Dulbecco’s Modified Eagle Medium (DMEM) (high glucose, 1.5 g/L NaHCO_3_) supplemented with 25,000 IU/L penicillin, 25 mg/L streptomycin, and 10% FCS (heat-inactivated) in 50 mL TubeSpin bioreactors. The tubes were kept in an incubator at 37 °C and 5% CO_2_ on a roller culture apparatus (5 rpm) [23]. The cells were passaged twice a week, diluting them in a ratio of 1:10. The macrophages were used until passage 30. To determine the cell count, the cell suspension was resuspended and diluted 1:2 with PBS. After fixation of the cells by mixing the cell suspension 1:1 with a formaldehyde solution (2%, *v*/*v*), aliquots of the resulting suspension were transferred into a standard Neubauer chamber (VWR, Austria) and counted manually using a light microscope (Motic BA310, Austria) at a magnification of 400×.

### 2.6. Resazurin Assay

LtP (2 × 10^6^ cells/mL) in a mixture of YEM and PBS (1:1, *v*/*v*) containing 25,000 U/L penicillin, 25 mg/L streptomycin, and 6 µM hemin (YEM/PBS) were prepared. Aliquots of 200 µL of this suspension were distributed in 96-well plates. Control rows containing YEM/PBS (no activity) and with untreated LtP (100% activity) were also loaded on the plate. To the wells of third rows, compounds (leading to a final vehicle concentration of max. 2% dimethyl sulfoxide (DMSO) in the wells) were added. Subsequently, five 1:5 serial dilution steps were performed. The plates were incubated at 26 °C for 48 h. Following the incubation, 50 µL of a resazurin solution in PBS were added to each well, giving a final concentration of 20 µM. After 4 h of incubation, the fluorescence was measured at 560 nm excitation and 590 nm emission using a plate reader (Perkin Elmer Enspire, Germany). The resazurin assays with J774 cells were performed in analogy to the LtP resazurin assays. Aliquots of a J774 cell suspension in DMEM supplemented with 25,000 U/L penicillin, 25 mg/L streptomycin, and 10% FCS (heat-inactivated), giving a final cell concentration of 10^5^ cells/mL, were distributed in the wells of the 96-well microplates. The cells were incubated for 24 h (37 °C, 5% CO_2_) to allow attachment. Then the medium was discarded and non-adherent cells were removed with 200 µL PBS per well. After the addition of DMEM, the compounds were added, and a serial dilution was performed. Control rows with DMEM (no activity) and with untreated J774 cells (100% activity) were also included. The plates were incubated again for 24 h at 37 °C and 5% CO_2_. Then the addition of resazurin and the measurement of fluorescence were carried out in analogy to the viability assays performed in LtP.

### 2.7. MTT Assay

Owing to the acidic conditions needed for axLtA (pH 4.5), viability assays with axLtA were carried out using MTT instead of resazurin that is unstable in an acidic environment. Cell suspensions (200 µL) of axLtA (6 × 10^6^ parasites/mL) in Schneider’s medium (pH = 4.5), containing 25,000 U/L penicillin, 25 mg/L streptomycin, and 50% FCS were prepared and distributed in 96-well round-bottom microplates. Then compounds were added (leading to a final vehicle concentration of max. 2% DMSO in the wells). Subsequently, five 1:5 serial dilution steps were performed. Control rows were loaded with 200 µL of either Schneider’s medium or axLtA suspension. After incubation at 32 °C for 48 h, 20 µL of MTT solution (in PBS) were added to each well (1 mM final concentration) and incubated for additional 4 h. Then plates were centrifuged at 400× *g* for 5 min (Hettich Rotina 420, Tuttlingen, Germany), and the supernatant was discarded. DMSO (100 µL) was added to each well and incubated at 32 °C for 10 min. Then the OD at 570 nm and 630 nm was determined using a plate reader (Perkin Elmer Enspire, Rodgau, Germany).

### 2.8. MTS Assay

The antileishmanial activity of anthracene endoperoxides (AcEPs) in LdP was established in promastigotes, and cell viability was measured using the modified MTS-PMS assay [24]. Briefly, log phase promastigotes (1 × 10^5^ cells/200 µL of Schneider‘s medium/well) were incubated with AcEPs (0–100 µM) for 48 h, and afterwards, parasite viability was measured. For this, MTS (2.0 mg/mL) and PMS (0.92 mg/mL) were added in a ratio of 5:1 (20 µL/well), and plates were incubated for 3 h at 37 °C; resultant absorbance was measured at 490 nm in a spectrofluorimeter (Merilyzer EIaQuant, Meril Life Sciences, Gujarat, India). The mean percent viability was calculated as follows: mean specific absorbance of treated parasites/mean specific absorbance of untreated parasites × 100. Results were expressed as IC_50_ values. These were enumerated by graphical extrapolation using Graph pad Prism software (version 5).

### 2.9. Xylenol Orange Assay

Xylenol orange (XO) (125 µM) and butylated hydroxytoluene (4 mM) were dissolved in methanol/H_2_O (9:1, *v*/*v*). FeSO_4_ (25 mM) in 2.5 M H_2_SO_4_ was prepared immediately before use and added, giving a final concentration of 250 µM FeSO_4_ in the samples. An aliquot of 100 µL EP solution (final concentration of 50 µM) was transferred into a disposable 1.5 mL cuvette (BRAND, Wertheim, Germany), and 1 mL of the color reagent was added. The samples that reacted slower (containing AcEP1117 or AcEP1130) were incubated for 0, 30, 60, and 90 min at 25 °C. At these time points, the OD difference of the sample at 560 nm and 650 nm was determined using a MS1501 UV-vis diode array spectrophotometer (Shimadzu, Japan). As reference, a mixture of methanol/H_2_O (9:1, *v*/*v*) was used [25]. The samples that reacted faster (containing AcEP1118 or AcEP1129) were scanned every 5 s over a time period of 5 min. The slope of the reaction rate was calculated using an extinction coefficient of the complex of 1.5 × 10^4^ M^−1^ cm^−1^.

### 2.10. Electron Paramagnetic Resonance (EPR) Spin Trapping: Activation of AcEPs by Fe^2+^

The reactions of AcEP1117, AcEP1118, AcEP1129, and AcEP1130 with Fe^2+^ were followed by electron paramagnetic resonance (EPR) spectroscopy. Therefore, an aliquot of purified water (MilliQ, Adavantage A10, Merck, Darmstadt, Germany) was mixed with the spin trap DMPO, giving a final concentration of 40 mM. Then the respective substance (stock solution in acetonitrile) was added to the sample, giving final concentrations of 500 µM of AcEP1117, AcEP1118, AcEP1129, or AcEP1130. To start the reaction, 500 µM FeSO_4_ (stock solution in purified water) were added. Samples containing 500 µM Ac, 200 µM anthraquinone (AQ), or a mixture of DMPO and FeSO_4_ in purified water alone were used as negative controls. After an incubation period of 5 min at 25 °C, the mixture was aspirated into a disposable glass micropipette (BLAUBRAND intraMARK, BRAND, Wertheim, Germany) and put into the split ring resonator (Bruker MD5) of the EPR spectrometer (Bruker EMX, Rheinstetten, Germany). The measurement was started with following parameters: microwave frequency 9.684 GHz; microwave power 20 mW, modulation frequency 100 kHz; modulation amplitude 2 G; time constant 0.164 s; center field 3446 G; scan rate 71 G/min; sweep width 100 G; scan time 84 s; attenuation 1.00 × 10^5^; and scans 1.

### 2.11. EPR Spin Trapping: Activation of AcEPs by LtP

To detect the radicals derived from AcEPs in LtP, EPR experiments were performed using DMPO as spin trap. Aliquots of the cell broth were transferred into Sarstedt tubes that were centrifuged (20 °C, 1800× *g*, 10 min), and the supernatants were discarded. Then the cell pellets were resuspended in PBS and centrifuged again. After removal of the supernatants, the cell pellets were resuspended in a mixture of PBS containing glucose (15 mM), giving a final cell number of 2 × 10^9^ LtP/mL. Aliquots (150 µL) were incubated in 1.5 mL Eppendorf tubes with aeration holes. The cells were incubated with DMPO (40 mM) for 5 min at 26 °C, and after addition of 500 µM of the respective substance (Asc, AcEP1117, AcEP1118, AcEP1129, AcEP1130, Ac or AQ), the samples were incubated for additional 15 min at 26 °C under constant agitation (agitation speed: 0.3 s^−1^). Control samples containing LtP, LtP and DMSO (1.27%, *v*/*v*), or PBS/glucose alone were incubated with 40 mM DMPO under the same conditions. Cell samples containing DMPO that were treated with Asc served as positive control. Samples treated with Ac or AQ were used as negative controls. Subsequently, 50 µL of the samples were aspirated into glass capillaries and transferred in the MD5 resonator of the EPR spectrometer. The samples were measured immediately using the following instrument settings: microwave frequency 9.684 GHz; microwave power 20 mW, modulation frequency 100 kHz; modulation amplitude 2 G; time constant 0.164 s; center field 3449 G; scan rate 71 G/min; sweep width 100 G; scan time 84 s; attenuation 8.93 × 10^5^; and scans 3.

### 2.12. EPR: Singlet Oxygen Formation

The potential release of singlet oxygen from anthracene endoperoxides (AcEPs) at 25 °C was followed by EPR spectroscopy using the spin trap TEMP. Aliquots of purified water containing DFO (100 µM) and DTPA (25 µM) were mixed with TEMP (50 mM) and the respective AcEPs (100 µM; AcEP1117, AcEP1118, AcEP1129 or AcEP1130). Samples containing TEMP (50 mM) and rose bengal (40 µM, RB) were used as positive control. After incubation for 5 min at 25 °C, aliquots (17 µL) of the respective samples were aspirated into a polytetrafluorethylene (PTFE) tube (outer diameter 0.7 mm). The tube was transferred into the MD5 resonator of the EPR spectrometer. Immediately, the measurement was started using following parameters: microwave frequency 9.684 GHz; microwave power 20 mW, modulation frequency 100 kHz; modulation amplitude 1 G; time constant 0.82 s; center field 3449 G; scan rate 14 G/min; sweep width 100 G; scan time 419 s; attenuation 1.00 × 10^4^; and scans 5. Measurements of the samples containing RB were carried out before and after irradiating the PTFE tube containing the sample with blue light (465 nm; LED Luxeon Star 3 W). Duration of irradiation was 15 min.

### 2.13. EPR: Superoxide Formation in LtP

To measure superoxide radical (O_2_^•−^) formation induced by AcEPs in LtP, EPR spectroscopy was used. An aliquot of the cell broth was transferred into an Eppendorf tube that was centrifuged (Hettich Universal 16, 20 °C, 2000× *g*, 10 min). The supernatant was discarded, the cell pellet was resuspended in PBS and centrifuged again. After removal of the supernatant, the cell pellet was resuspended in a mixture of PBS, 15 mM glucose (PBS/Glu), DFO (100 µM), and DTPA (25 µM). The cell suspension had a final cell number of 500 × 10^6^ cells/mL. The cell suspension was aliquoted. CMH was added as detection agent leading to a final concentration of 400 µM. After 5 min of incubation at 25 °C, the respective substance was added resulting in final concentrations of 20 µM antimycin A (AA) or 100 µM of AcEP1117, AcEP1118, AcEP1129, AcEP1130, Ac, and AQ. Samples containing LtP and CMH were used as negative control. Samples containing PBS/Glu and CMH were used as negative control as well. After 5 min of incubation at 25 °C with the respective substance, aliquots of 17 µL of the cell suspensions were aspirated into a PTFE tube (outer diameter 0.7 mm). The tube was transferred into the MD5 resonator of the EPR spectrometer. Immediately, the measurement was started using the following parameters: microwave frequency 9.684 GHz; microwave power 20 mW, modulation frequency 100 kHz; modulation amplitude 1 G; time constant 0.82 s; center field 3449 G; scan rate 14 G/min; sweep width 100 G; scan time 419 s; attenuation 2.52 × 10^4^; and scans 5.

### 2.14. O_2_ Consumption of LtP

Oxygen consumption rates of LtP treated with AcEPs were measured using round-bottomed OxoPlates (OP96U, Precision Sensing GmbH, Regensburg, Germany). An aliquot of BHI culture medium was transferred to an open Erlenmeyer flask and was stirred on a magnetic stirrer for 30 min at 25 °C to saturate it with air (aBHI). Another aliquot of BHI culture medium was mixed with approximately 50 mg of the reducing agent sodium dithionite in a tightly closed falcon tube to remove the dissolved oxygen (dBHI). Aliquots of 200 µL of both calibration media were distributed to 6 wells each of the first row of an OxoPlate. To prevent oxygen exchange with the environment, 70 µL of paraffin oil were layered on top of each well containing liquid. The plate was then incubated in the dark for 15 min at 25 °C for equilibration. Then the fluorescence of both calibration media was measured at a microplate reader (Perkin Elmer Enspire, Germany) in a dual kinetic mode using an excitation wavelength of 540 nm for both of the dyes, emission wavelengths of 650 nm for the indicator dye, and 590 nm for the reference dye. The ratio of both intensities (I_R_) was calculated for each well and averaged giving the two calibration constants k_0_ or k_100_. From the second row on, each well was filled with 50 µL of aBHI. The third row was provided with extra 50 µL of aBHI. The respective amounts of the anthracene derivatives (Ac, AQ, AcEP1117, AcEP1118, AcEP1129, AcEP1130) were added and mixed properly. A serial dilution was performed by transferring 50 µL of the mixture in five subsequent 1:2 steps. LtP (140 × 10^6^ cells/mL) were suspended in aBHI and aliquots of 150 µL were transferred to each well giving a maximal final concentration of the AcEPs of 200 µM, declining with onward 1:2 dilution steps. On top of each well, 70 µL of paraffin oil was added. The fluorescence intensity of each well was determined in 5 min intervals over 40 min. Each concentration was measured in quadruplicates. The oxygen concentration was calculated using the following equation (PreSens–Precision Sensing GmbH):

O_2_ [µM] = 100 × (k_0_/I_R_ − 1) / (k_0_/k_100_ − 1) × 2.83

The slope of the change of the oxygen concentrations over time was calculated giving the oxygen consumption rate [µM/min] for each well. The value obtained for aBHI was subtracted to eliminate the influence of the medium. The rate was finally normalized to the oxygen consumption rate of the wells that did contain cells but no test compounds.

### 2.15. Uncoupling of LtP Respiration

The uncoupling experiments using OxoPlates were carried out in analogy to oxygen consumption measurements of LtP. An oligomycin (Oligo) premix in aBHI was prepared leading to a final concentration of 38 µM oligomycin (Oligo). Each well of the second row was filled with 50 µL of aBHI. From the third row on, each well was filled with 50 µL of the Oligo premix and to the fourth row additional 50 µL Oligo premix was added. The respective amounts of the substances (Ac, AQ, AcEP1117, AcEP1118, AcEP1129, AcEP1130, CCCP) were added and a serial 1:2 dilution was performed. Measurements were carried out as described above. The oxygen consumption rates were normalized to the wells that contained only LtP. For each concentration measurements were done in triplicates.

### 2.16. Data Analysis

Calculations were carried out using Microsoft Excel (Microsoft). The graphs were created using Origin 6.1 (OriginLab Corporation, Northampton, MA, USA). The half maximal inhibitory concentrations (IC_50_) were determined from non-linear concentration–response curves using a four-parameter logistic model and expressed as the mean ± standard deviation (SD). Statistically significant differences of *p* <0.05 were identified using Students t-test.

## 3. Results

**Figure 1:**
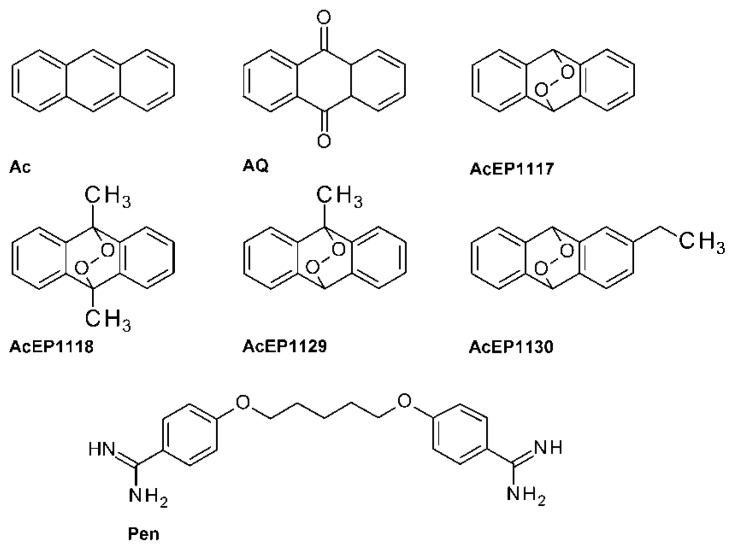
Structures of anthracene endoperoxides (AcEPs) and other compounds (Ac, anthracene; AQ, anthraquinone; Pen, pentamidine) used in this study.

To study the potential of AcEPs as antileishmanial drugs and structure-related effects within this group (Figure 1), we compared their activity to the non-peroxide analogs anthracene (Ac) and anthraquinone (AQ). Furthermore, pentamidine (Pen) was included as an antileishmanial reference compound.

### 3.1. Effects of AcEPs on Cell Viability

The influence of AcEPs and reference compounds on cell viability (as IC_50_) was investigated using resazurin- and tetrazolium-based assays in *Leishmania tarentolae* promastigotes (LtP), *Leishmania donovani* promastigotes (LdP) and J774 macrophages. In axenic *Leishmania tarentolae* amastigotes (axLtA), a MTT-based assay was used. As indicated in Table 1, Pen showed IC_50_ values below 1 µM in LtP, LdP, and axLtA, endorsing its potent antileishmanial activity. In J774 macrophages, Pen was less cytotoxic, corroborating its selectivity. The AcEP compounds compared to the non-peroxidic Ac and AQ showed lower IC_50_ values in all cells. Some AcEPs demonstrated IC_50_ values lower than Pen in *Leishmania*. AcEPs with structures containing an alkyl substituted anthracene moiety (AcEP1118, AcEP1129 and AcEP1130) exhibited lower IC_50_ values in LtP. However, in general AcEPs also showed some toxic effects on J774 cells. Nevertheless, comparing the IC_50_ values in all *Leishmania* strains to the corresponding values in J774 macrophages, a limited selectivity of some AcEPs for *Leishmania* was revealed.

To explore the antileishmanial mechanism of AcEPs, we studied their activation and resulting consequences in *Leishmania* in a chemical system as well as in LtP.

### 3.2. Reactivity of AcEPs with Fe^2+^

In a biomimetic approach, we compared the reactivity of AcEPs and other compounds with Fe^2+^, which was detected by simultaneous complexation of the formed Fe^3+^ by XO. Accordingly, the photometric detection of the XO/Fe^3+^ complex formation over time provides a measure for the reaction of the EP with Fe^2+^. The slopes of the XO/Fe^3+^ complex formation (Table 2) for EPs in the presence of Fe^2+^ show largely different rates. The antileishmanial EP Asc showed greater rates than Art. However, the endoperoxides AcEP1118 and AcEP1129 showed a clearly enhanced formation rate of the XO/Fe^3+^ complex and therefore a high reactivity with Fe^2+^. Specifically, AcEP1129 was 300 times more reactive than Asc in this assay.

### 3.3. Radical Formation from AcEP Activation by Fe^2+^

**Figure 2:**
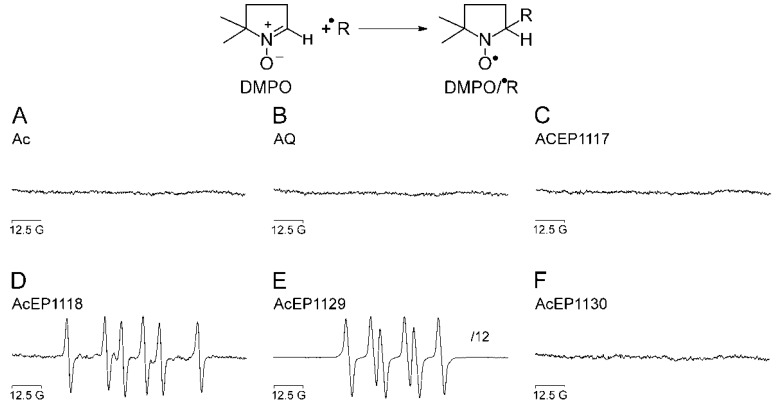
EPR spectra recorded after incubation of AcEPs and other compounds with Fe^2+^ in the presence of the spin trap DMPO. The spectra were obtained after an incubation of 5 min of 500 µM FeSO_4_ with (**A**) 500 µM anthracene (Ac), (**B**) 200 µM anthraquinone (AQ), (**C**) 500 µM AcEP1117, (**D**) 500 µM AcEP1118, (**E**) 500 µM AcEP1129 (intensity scaled-down by a factor of 12), or (**F**) 500 µM AcEP1130 in the presence of 40 mM DMPO in H_2_O at 25 °C. The graph shows representative spectra of three independent experiments.

In a next step, we measured the radical formation from the reaction of Fe^2+^ and AcEPs in H_2_O in the presence of 5,5-dimethyl-1-pyrroline-N-oxide (DMPO) as spin trap using EPR spectroscopy. After 5 min incubation of the reaction mixture, the EPR spectra shown in Figure 2 were obtained.

Compounds not containing a peroxide group (Ac and AQ) did not show EPR signals of spin adducts under these experimental conditions (Figure 2A,B). Likewise, AcEP1117 and AcEP1130, although they are EPs, did not result in detectable DMPO adducts (Figure 2C,F). In contrast, AcEP1118 and AcEP1129 showed six-line EPR spectra, however, with different splitting patterns (Figure 2D,E). For AcEP1129, the EPR signal intensities were higher than for AcEP1118. Simulations of the obtained spectra for AcEP1118 revealed coupling constants of a_N_ = 16.3 G and a_H_ = 23.2 G, suggesting the trapping of a carbon-centered radical [26,27]. In contrast, EPR signals derived from AcEP1129 activation gave coupling constants of a_N_ = 14.4 G and a_H_ = 10.5 G, which were compatible with organic peroxyl radicals as primary products [28].

### 3.4. Radical Formation from AcEPs in Leishmania

**Figure 3:**
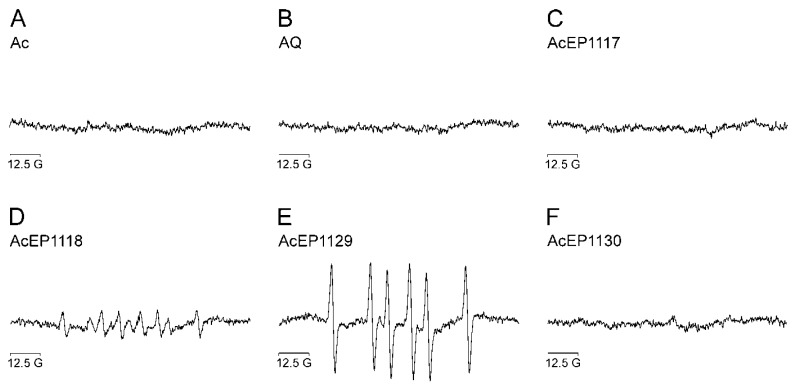
EPR spectra obtained after activation of AcEPs in *Leishmania* in the presence of the spin trap DMPO. The spectra shown were obtained after incubation of LtP (2 × 10^9^ LtP/mL) for 15 min with 500 µM of (**A**) anthracene (Ac), (**B**) anthraquinone (AQ), (**C**) AcEP1117, (**D**) AcEP1118, (**E**) AcEP1129 or (**F**) AcEP1130 in the presence of 40 mM DMPO. The maximum concentration of the vehicle (DMSO) was 1.27%. Reactions were performed in a mixture of PBS and glucose (15 mM) at 25 °C. The graph shows representative spectra of three independent experiments.

To verify the relevance of these findings from a biomimetic system in *Leishmania* we performed analogous experiments in LtP cells without the addition of iron (Figure 3). The EP Asc showed DMPO spin adducts of carbon-centered radicals with coupling constants of a_N_ = 16.4 G and a_H_ = 24.9 G (spectra not shown). Under these experimental conditions (short incubation, intermediate DMPO concentrations, no iron chelators), neither Ac nor AQ resulted in detectable DMPO spin adducts (Figure 3A,B). Likewise, no spin adducts were observed for AcEP1117 and AcEP1130 in LtP (Figure 3C,F). In LtP, upon incubation with AcEP1118, a moderate intensity DMPO spin adduct was observed (Figure 3D). Spectral simulations revealed coupling constants of a_N_ = 16.2 G and a_H_ = 23.4 G (major component), which are similar to the coupling constants in the biomimetic system. This suggested the trapping of carbon-centered radicals from AcEP1118 in both systems [29]. For AcEP1129 in LtP, high intensity EPR spectra of spin adducts were observed (Figure 3E). Simulations revealed coupling parameters for the obtained spectra of AcEP1129 of a_N_ = 16.3 G and a_H_ = 23.3 G, suggesting carbon-centered radicals [30] as the result of the reaction in contrast to the biomimetic system in which peroxyl radicals were trapped from AcEP1129.

### 3.5. Potential Release of ^1^O_2_ from AcEPs

**Figure 4:**
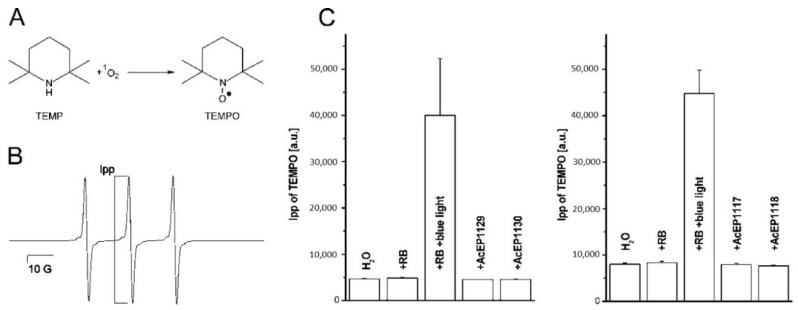
Peak-to-peak intensity (Ipp) of the EPR signals resulting from TEMP oxidation by ^1^O_2_. (**A**) Scheme of the reaction of TEMP with ^1^O_2_ resulting in the formation of TEMPO. (**B**) Singlet oxygen reacts with TEMP to TEMPO, a stable nitroxyl radical, resulting in a three-line spectrum. (**C**) Each of 100 µM of AcEP1117, AcEP1118, AcEP1129, or AcEP1130 in H_2_O were incubated for 5 min at 25 °C in the presence of 50 mM TEMP. Samples containing 50 mM TEMP and rose bengal (40 µM, RB) served as the positive control. Samples containing 50 mM TEMP in H_2_O only were used as negative control. The positive control was measured in addition after irradiating the samples with blue light (465 nm) for 15 min. Data represent mean ± SD (*n* = 3).

In a next step, we studied the potential release of ^1^O_2_ from AcEPs under thermal conditions (25 °C) in a biomimetic system. To detect ^1^O_2_ released under these conditions, we used TEMP [31,32]. Upon addition of ^1^O_2_ to the hydroxylamine TEMP (2,2,6,6-tetramethylpiperidine), the stable nitroxyl radical (2,2,6,6-tetramethylpiperidine-1-yl)oxyl (TEMPO) (Figure 4A) is formed, which can be detected as a three-line EPR spectrum (Figure 4B; a_N_ = 17.3 G). The peak-to-peak intensity of the middle peak (Ipp) is proportional to the amount of TEMPO. Spectra were measured after 5 min of incubation of AcEPs with TEMP.

Samples containing TEMP and RB after irradiation with blue light were used to confirm the suitability of the method to detect the formation of ^1^O_2_. As can be seen (Figure 4C), in this system RB triggers a boost of TEMPO formation upon blue light irradiation, and therefore, the release of ^1^O_2_. EPR experiments using TEMP as a probe for ^1^O_2_ demonstrated that under our thermal conditions (25 °C), studied AcEPs do not release significant amounts of ^1^O_2_ in this time scale.

### 3.6. Formation of Superoxide Radicals Triggered by AcEPs in Leishmania

**Figure 5:**
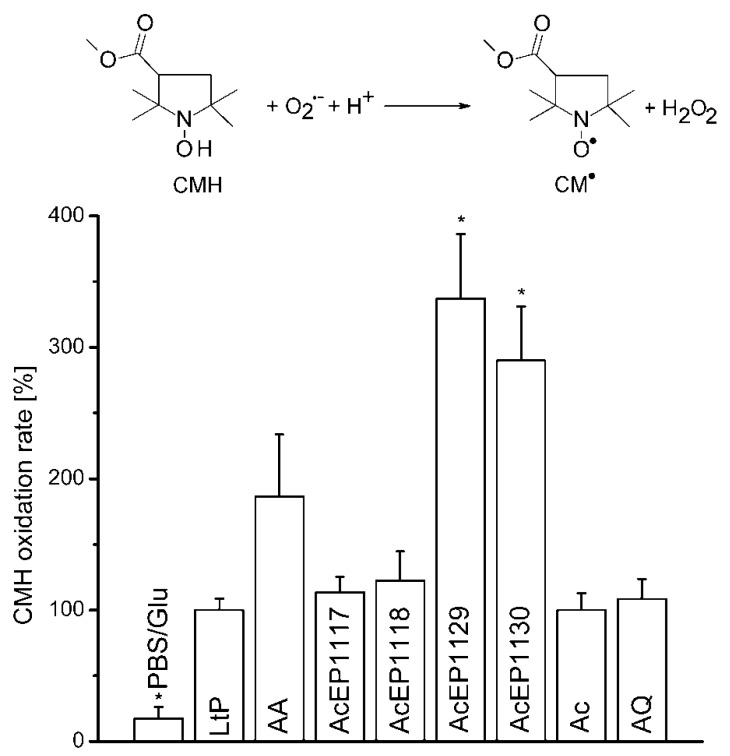
Rate of CMH oxidation by superoxide radicals derived from the activation of AcEPs in LtP. LtP (500 × 10^6^ LtP/mL) suspended in PBS containing glucose (15 mM, PBS/Glu), DFO (100 µM), DTPA (25 µM), and CMH (400 µM) were incubated with 20 µM AA or 100 µM of AcEP1117, AcEP1118, AcEP1129, AcEP1130, Ac, or AQ at 25 °C for 5 min. Negative controls consisted of PBS/Glu or cell suspension containing CMH. Data were normalized against the untreated cells (LtP, 100%). Data represent mean ± SD (*n* = 3), * indicates significant differences to the control sample (LtP) at the level of *p* < 0.05.

CMH was used as a detection system to evaluate the formation of O_2_^•−^ in cellular environments [33]. We used this method to investigate a potential contribution of superoxide radicals to the antileishmanial action of AcEPs. CMH gets oxidized by superoxide radicals forming stable nitroxyl CM^•^ (Figure 5) that can be detected by EPR spectroscopy. This activity was examined in *Leishmania* parasites in the presence of AcEPs and other compounds (Figure 5).

Experiments with the CMH detection system were performed in the presence of iron chelators (DFO, DTPA) to prevent interference by Fe^3+^-mediated CMH oxidation. In LtP, basal CMH oxidation was higher than in PBS (Figure 5). Antimycin A (AA), a known initiator of mitochondrial superoxide formation, triggered additional CMH oxidation in LtP. Ac and AQ did not trigger additional CMH oxidation in LtP under our conditions. For the AcEPs, an increased CMH oxidation rate occurred with AcEP1129 and AcEP1130, whereas AcEP1117 and AcEP1118 failed to cause any enhanced oxidation of CMH (Figure 5). To exclude the spontaneous release of superoxide radicals from AcEPs, control experiments without cells were performed, which showed no enhancement of the CMH oxidation in the presence of AcEPs (data not shown).

### 3.7. Influence of AcEPs on O_2_ Consumption Rate of Leishmania

To verify the involvement of leishmanial mitochondria in AcEP actions, the aerobic energy metabolism of LtP cell suspensions in PBS/Glu was studied in 96-well plates with oxygen-sensitive fluorescence sensors (OxoPlates, PreSens, Germany). The influence of AcEPs, Ac, and AQ on LtP respiration was tested using concentrations ranging from 6.25–200 µM.

**Figure 6:**
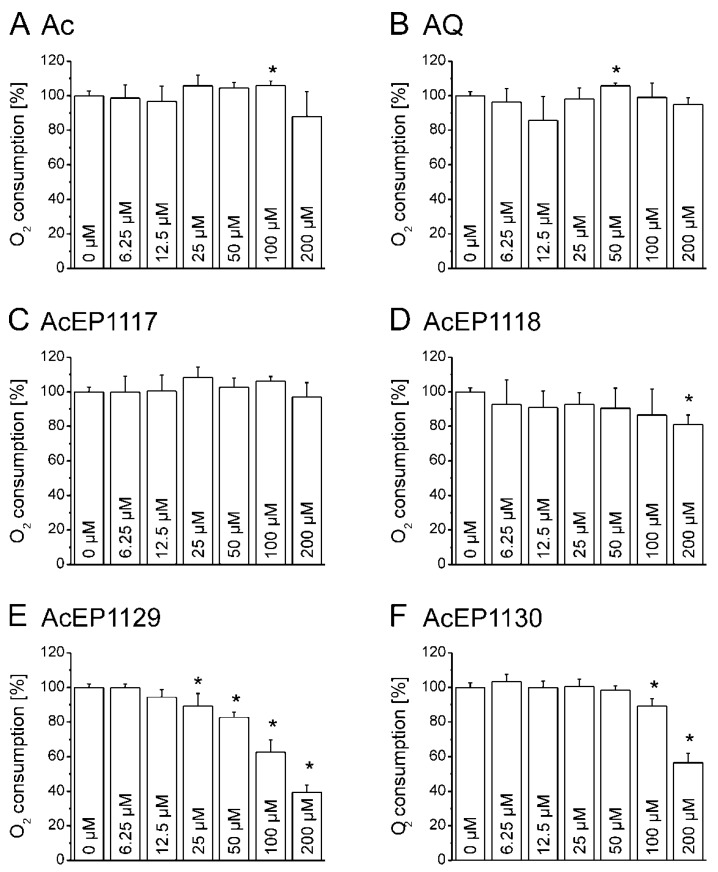
Influence of different anthracene derivatives on the oxygen consumption of LtP. LtP (140 × 10^6^ cells/mL) were treated with 6.25 to 200 µM of Ac (**A**), AQ (**B**), AcEP1117 (**C**), AcEP1118 (**D**), AcEP1129 (**E**) or AcEP1130 (**F**) (max. vehicle concentration 1.5% DMSO). Untreated LtP in BHI medium served as reference (0 µM, 100%). Measurements were carried out using OxoPlates over an incubation time of 40 min. Data represent mean ± SD (*n* = 3–4), * indicates significant differences to the control samples (0 µM) at the level of *p* < 0.05.

The non-endoperoxides Ac and AQ served as negative controls and showed no considerable influence on the LtP respiration up to concentrations of 200 µM as did the endoperoxides AcEP1117 and AcEP1118 (Figure 6). In contrast, the AcEPs 1129 and 1130 showed a significant decline in the oxygen consumption rate at higher concentrations. The effect was stronger for AcEP1129, which mediated a significant decrease in oxygen consumption beginning at concentrations of 25 µM. To exclude the possibility of an oxygen release by AcEPs, experiments were repeated excluding cells. It was shown that the AcEPs had no effect on oxygen levels in a cell-free system (data not shown).

### 3.8. Influence of AcEPs on Mitochondrial Coupling in Leishmania

**Figure 7:**
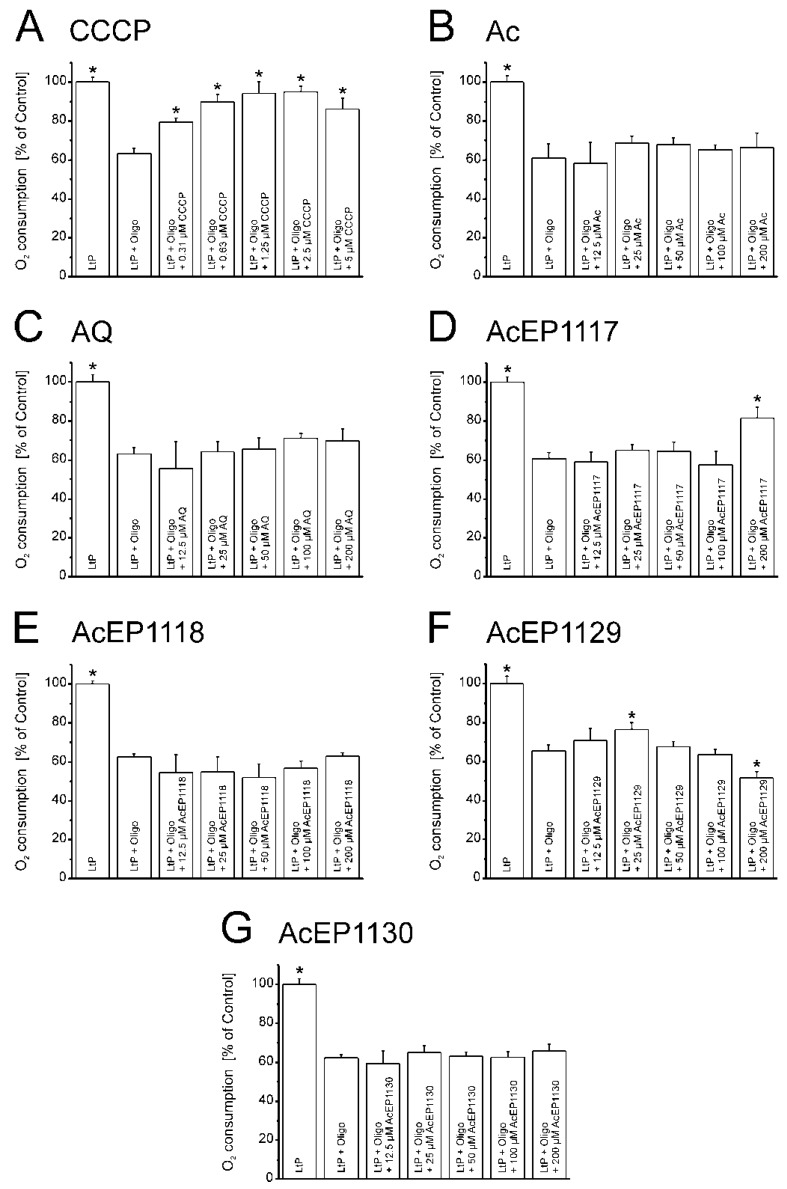
Influence of different anthracene derivatives on the oxygen consumption of LtP in the presence of the ATPase inhibitor oligomycin (Oligo, 38 µM). LtP (140 × 10^6^ cells/mL) were treated with 0.31–5.0 µM CCCP (**A**) or 12.5–200.0 µM of Ac (**B**), AQ (**C**), AcEP1117 (**D**), AcEP1118 (**E**), AcEP1129 (**F**), AcEP1130 (**G**) (max. vehicle concentration 1.5% DMSO). Samples containing LtP in BHI medium with and without Oligo served as control. Measurements were carried out using OxoPlates over an incubation time of 40 min. Data were normalized against untreated cells (LtP, 100%). Data represent mean ± SD (*n* = 3–4), * indicates significant differences to the control samples (LtP + Oligo) at the level of *p* < 0.05.

Besides the inhibition of mitochondrial electron transfer, another vulnerable process in *Leishmania* is the chemiosmotic coupling between the mitochondrial proton gradient and ATP synthesis. To assess the mitochondrial coupling in LtP, additional experiments using OxoPlates were carried out. However, in these experiments, the possible stimulation of oxygen consumption by AcEPs in the presence of oligomycin (Oligo), an inhibitor of ATP synthase, was studied (Figure 7).

Treating LtP with Oligo reduced the oxygen consumption rate to about 60% compared to untreated LtP. This suggests that about 40% of oxygen consumption in LtP is used for ATP production. As expected, in the presence of the uncoupler CCCP (Figure 7A), a recovery of O_2_ consumption in the presence of Oligo was observed. However, the recovery remained below the respiration of untreated cells reflecting the absence of any spare capacity in LtP. Neither Ac nor AQ displayed an uncoupling effect under these conditions (Figure 7B,C). For AcEPs (Figure 7D–G) no strong uncoupling was observed except for AcEP1117 at 200 µM (Figure 7D), and for AcEP1129 (Figure 7F), a trend for uncoupling was observed at lower concentrations, which at higher concentrations, was overridden by its inhibiting properties seen in Figure 6E.

## 4. Discussion

Therapy of parasitic diseases such as leishmaniasis involves serious difficulties as the complicated and complex life-cycle of the pathogens, constant development of resistances against established treatment options, and the tolerability of the remedies causing severe side effects. Therefore, alternative therapy options and a continuous adaption to the constantly evolving pathogens are not only urgently needed, but desperately searched for.

Such an alternative option for the treatment of parasitic infections caused by unicellular pathogens, such as malaria is the EP artemisinin (Art). The plant-derived sesquiterpene and EP is used as single compound or as component of Art-based combination therapies against infections with *Plasmodia*. Previous studies also showed a strong effect of Art against the unicellular protozoal parasites of the genus *Leishmania* [12,13,16].

Another example for a plant-derived EP with a strong antiparasitic effect is the monoterpene Asc that showed a profound activity against *Leishmania* as well. Previous studies demonstrated its effectivity in a cutaneous leishmaniasis model established in *L. amazonensis*-infected mice [34]. Nevertheless, differences in pharmacological and pharmacokinetic properties of Art and Asc due to their structural divergence raised the interest of testing other EPs with various structures to elucidate their effectivity against *Leishmania*.

AcEPs consist of a basic anthracene moiety with an attached endoperoxide bridge and other variable substituents (Figure 1). From a pharmacological point of view, AcEPs are interesting substances due to their potential role as singlet oxygen (^1^O_2_) carriers/donors. It was demonstrated that the cleavage of the endoperoxide bridge by thermal stimulation [35,36] or light-induced photo activation [37] leads to the release of the potent oxidant ^1^O_2_ by some AcEPs. Schmidt and Brauer have shown that for many endoperoxides under thermal conditions, the rates for peroxide bond breakage and for cycloreversion, i.e., release of ^1^O_2,_ is in the same order of magnitude [38]. In technical applications, the AcEPs are therefore used as photoresist or photoswitch for lithography [17].

The potential release of ^1^O_2_ and other reactive oxygen species under thermal, photochemical, and other conditions by AcEPs suggests their use as potential agents against *Leishmania.*

Hence, this study aimed at verifying the antileishmanial potential of the four synthetic AcEPs 1117, 1118, 1129, and 1130 as well as their mechanism with respect to intermediates and influence on leishmanial mitochondria.

### 4.1. Influence of EPs on Viability

Studies from other authors demonstrated the antileishmanial potency of EPs [12,39]. In previous studies, we focused on Asc and Art and investigated the origin of their action against *Leishmania* [11,16,40]. It was evident that radicals, namely isopropyl radicals for Asc and more complicated carbon-centered radicals for Art, play a major role in their mode of action [16]. However, in this study, we examined EPs based on an anthracene structure (Figure 1) and if differences in their substitution pattern lead to variation in their potential.

Therefore, resazurin- and tetrazolium-based assays were used in LtP, LdP, and J774 macrophages to explore the effects of the AcEPs on the viability of the parasites and their mammalian host cells (Table 1). For axLtA, which require acidic culture media, a MTT assay was used, because resazurin was not stable under these conditions. Besides the AcEPs, Pen, an established antileishmanial drug, Ac and AQ, which are structurally related to the AcEPs but lacking the endoperoxide bridge, were used as reference compounds.

As expected, Pen showed a high antileishmanial effect for LtP, LdP, and axLtA, whereas its toxicity in J774 macrophages was moderate. In general, the AcEPs showed IC_50_ values for LtP and axLtA in low micromolar concentrations with slightly higher values in axLtA. This could be due to the lower proliferation rate of axLtA vs. LtP. In pathogenic *Leishmania* (LdP), IC_50_ values were similar to values of LtP (except for AcEP1130). The IC_50_ values in LtP were lower than in the macrophages. They indeed had a toxic effect on J774 too, but showed a slight preference towards killing the parasites. Especially the AcEPs with alkyl substituents were more effective than the unsubstituted ones in both forms of *L. tarentolae*. Ac and AQ showed considerably higher IC_50_ values than AcEPs, indicating a mechanistic relevance of the EP group. This observation is in qualitative agreement with the comparison of Asc with 1,4-cineole, which share a similar structure, but only Asc has the endoperoxide group. Also, in this case, Asc was much more active against LtP than 1,4-cineole [16]. This is especially interesting for AQ, presenting a degradation product of AcEPs [41], and confirms that AQ upon formation does not contribute significantly to the antileishmanial action of the AcEPs.

Despite the fact that the AcEPs showed promising antileishmanial potency, their selectivity for the parasites has to be enhanced. However, that such problems can be overcome in principle has been shown already for the antileishmanial remedy AmBisome which minimizes host toxicity of amphotericin B by liposomal encapsulation [42]. Other possible approaches to enhance the selectivity of AcEPs include different substitution patterns for AcEPs with other alkyl substituents, improved targeting by specific liposomes, or site-directed activation by heat or light. However, an essential prerequisite for optimization is to gain insights in the mechanism of action of AcEPs.

### 4.2. Studied AcEPs Do Not Release ^1^O_2_

From the chemistry of AcEPs in general [20], it is known that they can reversibly release ^1^O_2_ and hydrogen peroxide [43]. The release of singlet oxygen from the AcEPs can be triggered by thermal induction [20] or exposing the EP to UV light (100 nm–380 nm) [41].

Therefore, we followed the formation of singlet oxygen originating from the AcEPs in a chemical buffer system at room temperature (Figure 4). EPR experiments were carried out using TEMP as spin trap for ^1^O_2_ forming thereby the stable nitroxyl radical TEMPO visible as a triplet in the EPR spectrum (Figure 4A,B) [32]. The well-known photosensitizer RB gave a TEMPO signal without illumination at the level of H_2_O, however, a strongly increased signal upon irradiation with blue light (Figure 4C). The TEMPO signal intensities in the presence of AcEPs without illumination were at the levels of the corresponding H_2_O controls. This illustrated that the studied non-substituted or alkyl-substituted AcEPs do not release significant amounts of ^1^O_2_ under our thermal conditions (25 °C). The ability to release ^1^O_2_ from these compounds may strongly depend on their molecular structure. Kinetic measurements of acene EP decomposition by Linker and Fudickar revealed that in compounds that are non-substituted, or have alkyl or alkenyl residues attached to the bridgehead carbon atom (adjacent to the peroxo-bridge), preferably undergo O-O bond cleavage while aryl and alkynyl residues favor cycloreversion and liberation of ^1^O_2_ [44]. These data together with literature findings suggest that for studied AcEPs under our conditions, ^1^O_2_ is not involved in *Leishmania* killing.

### 4.3. Reactivity of AcEPs with Fe^2+^

Previous studies in biomimetic systems demonstrated that the endoperoxide bridge of EP needs to be reductively cleaved to unfold their pharmacological action [15,16,45]. This was also proven in *Leishmania* cells [16]. A major activator of EPs in the biological system is Fe^2+^ from the labile iron pool in *Leishmania*. Therefore, the reaction rate of AcEPs with Fe^2+^ was of interest. In a XO assay, we followed the formation of Fe^3+^ upon reduction of the EP by Fe^2+^ and subsequent XO/Fe^3+^ complex formation. The antileishmanial EPs Asc and Art showed rates (Asc > Art) which confirmed previously published data [16] (Table 2). AcEP1118 and especially AcEP1129 showed a rapid formation of Fe^3+^ upon reaction of Fe^2+^ with the respective EP. The reactivity of the AcEPs with Fe^2+^ in this experiment was even higher than that for Asc which was used for comparison purposes. This demonstrates that Fe^2+^ is an important reaction partner and could be a potential trigger of activation especially for AcEP1118 and AcEP1129 in *Leishmania*.

### 4.4. Radical Formation from AcEP Activation by Fe^2+^

The reaction of EPs with Fe^2+^ results in the reductive cleavage of their peroxide bridge and the subsequent formation of oxygen-centered radicals, which, however, depending on the molecular structure, break down to more stable carbon-centered radicals. This was shown in previous studies for Asc and Art [14,15,16]. To study the activation of AcEPs by Fe^2+^ and resulting products, EPR /DMPO spin trapping experiments in a chemical system were performed (Figure 2).

Our experiments demonstrated that the substances containing an anthracene structure but lacking the peroxide bridge did not result in the formation of detectable radicals in our system (Figure 2A,B). Also for AcEP1117 and AcEP1130, no signals of spin adducts were obtained under chosen conditions (Figure 2C,F). However, for AcEP1118 as well as for AcEP1129, six-line EPR spectra were observed (Figure 2D,E). For the reaction of AcEP1118 with Fe^2+^, the splitting patterns suggested the formation of carbon-centered radicals [26], whereas for AcEP1129, coupling constants suggested the formation of organic peroxyl radicals [28]. The formation of different radical species may indicate a structure-related effect upon cleavage of the peroxide bridge.

Based on studies of the reaction mechanism by Linker and Fudickar [20], the electron transfer from Fe^2+^ to AcEP1118 (dimethylated derivative) results in an alkoxyl radical. This initial intermediate then, however, extrudes a methyl radical from the molecules [46]. The formation of DMPO/^●^C adducts with AcEP1118 confirms this observation partially, while DMPO may not be sufficiently reactive to trap the alkoxyl intermediate under our conditions. The trapping of peroxyl radicals derived from AcEP1129 by DMPO suggests that asymmetric substitution of the peroxo bridgehead carbon atoms may influence the reaction mechanism. Again, the fact that for AcEP1118 and AcEP1129, DMPO radical adducts were observed fits to their high reactivity in the xylenol orange assay.

### 4.5. Radical Formation from AcEPs in Leishmania

Since LtP contain large quantities of Fe^2+^ in the so-called labile iron pool [16], it was of interest whether the spin adducts observed in the chemical system also can be observed in *Leishmania*. Therefore, EPR spin trapping experiments with AcEPs and LtP without addition of iron were carried out (Figure 3). Our EPR data demonstrated that in LtP, anthracenes lacking the EP bridge did not result in detectable spin adducts (Figure 3A,B). Also, AcEP1117 and AcEP1130 did not result in the formation of detectable radicals under our conditions (Figure 3C,F). AcEP1118, on the other hand, gave six-line spectra with coupling constants similar to the parameters obtained in the chemical system (a_N_ = 16.2 G and a_H_ = 23.4 G), suggesting the formation of carbon-centered radicals in the biological system as well (Figure 3D). For AcEP1129, on the other hand, the constants differed from the values that were acquired in the chemical system (a_N_ = 16.3 G and a_H_ = 23.3 G). The parameters resembled the coupling constants obtained for AcEP1118, suggesting the formation of carbon-centered radicals for AcEP1129 in the biological system (Figure 3E).

Detecting oxygen-centered radicals for AcEP1129 in the chemical system but carbon-centered radicals in *Leishmania* may be caused by the fast decline of the oxygen-centered AcEP1129 radicals in a surrounding that provides a variety of reaction partners which are available in the cell but not in the chemical system. In addition, electron transfer to AcEP1129 in cells can not only occur via low molecular Fe^2+^ complexes but also via heme compounds which may influence the actual reaction mechanism.

Again, spin adduct signals were just obtained for the AcEPs that showed the highest reaction rates in the XO assay before. This suggests that AcEP-derived radicals could play a major role in the pharmacological mechanism of AcEPs in *Leishmania*. Spin adducts were not detected for all AcEPs under our conditions, which might be due to the fact that for slowly reacting AcEPs (Table 2), an enrichment of spin adducts above the detection limits is not achieved.

### 4.6. CMH Oxidation Triggered by AcEPs in Leishmania

Therefore, we rather focused on downstream mechanisms of radical formation and damage by AcEPs in *Leishmania*. A key event in oxidative damage by drugs is the formation of superoxide radicals either as a result of drug metabolism or the damage of oxygen-metabolizing organelles in *Leishmania* [47,48]. For some AcEPs, we demonstrated the formation of carbon- and oxygen-centered radicals upon activation. It is known that in aerobic cellular environments, carbon-centered radicals finally react with oxygen to peroxyl radicals [49,50]. Peroxyl radicals can release ^1^O_2_ by the Russel mechanism [51] or could damage mitochondria, thereby triggering mitochondrial O_2_^•−^ release [52]. Since superoxide radicals are the starting point of the oxidative stress cascade, which causes severe oxidative damage to the cell [53], the contribution of O_2_^•−^ to the antileishmanial effect of the AcEPs was of interest.

We have not detected ^1^O_2_ originating from the studied AcEPs without LtP under room temperature conditions. To observe the O_2_^•−^ formation upon treating LtP with AcEPs, EPR measurements in the presence of the hydroxyl amine CMH, which reacts with O_2_^•−^ resulting in CM^•^, a stable nitroxyl radical, were carried out (Figure 5). The experiments with AcEPs revealed that compounds with asymmetric alkyl substitution (AcEP1129 and AcEP1130) resulted in a remarkable additional CMH oxidation, which could indicate increased formation of superoxide radicals or theoretically direct interaction of AcEP-derived radicals with CMH. In contrast, AcEP1117 and AcEP1118 as well as the reference substances Ac and AQ did not show such an effect. Control experiments without cells elucidated that the substances alone did not lead to an enhanced CMH oxidation (data not shown). To exclude a direct oxidation of CMH by the reaction with AcEP-derived carbon-centered radicals, we performed DMPO spin trapping experiments in LtP (as shown in Figure 3) but in the presence of 400 µM CMH. Under these conditions, intensities of six-line EPR spectra of carbon-centered radicals were not diminished excluding CMH as direct reactant of these carbon-centered radicals (data not shown). This suggests that the additional O_2_^•−^ are not spontaneously released from AcEPs themselves and exclude AcEPs as a direct source of superoxide radicals. The fact that we did not observe DMPO/O_2_^•−^ adducts in LtP with AcEPs could be based on the short half-life of these adducts compared to DMPO/^●^C adducts or that O_2_^•-^ is formed secondary to carbon-centered radicals, which are trapped by DMPO [54,55].

Additional O_2_^•−^ could originate from mitochondrial electron carriers upon damage by AcEP-derived radicals or cellular reductases. Furthermore, mitochondria could also play a role in the reductive activation of AcEPs.

### 4.7. Influence of AcEPs on O_2_ Consumption of Leishmania

Mitochondria in *Leishmania* are essential for parasite survival since only one of these organelles exists per cell, and they do not possess a respiratory spare capacity to respond to cellular stress. Therefore, it was of interest to elucidate whether the antileishmanial effect of AcEPs is partially mediated by mitochondrial impairment in *Leishmania*. The impairment of the mitochondrial electron transfer and coupling to phosphorylation has an impact on the cellular respiration. Damaging the leishmanial mitochondrion as a target of EP activation, hence, was expected to influence the oxygen consumption of LtP. The influence of the AcEPs and the reference substances Ac and AQ on the cellular oxygen consumption of LtP was investigated (Figure 6). Our experiments demonstrated that AcEP1130 and especially AcEP1129 reduced the oxygen consumption by LtP at higher concentrations, indicating an interference with the cellular respiration of LtP (Figure 6E,F) and an interference of these compounds with mitochondrial functions.

Considering the possibility of an oxygen release from AcEPs upon degradation of the peroxide bridge [41], the experiments were repeated without cells to examine the effect of the AcEPs on the oxygen levels (data not shown). These measurements demonstrated that under our conditions, AcEPs did not liberate or consume significant amounts of O_2_ without cells in this assay.

### 4.8. Influence of AcEPs on Mitochondrial Coupling in Leishmania

Another sensitive mitochondrial function is the coupling of electron transfer to phosphorylation. Some substances, such as the uncoupler CCCP, interfere with this mechanism by shuttling protons through the otherwise proton-tight inner mitochondrial membrane. This allows a persisting CCCP-dependent cellular respiration which is not coupled to phosphorylation [53,56]. This could also be expected under severe oxidative stress leading to lipid peroxidation in the inner mitochondrial membrane, which impairs its proton impermeability. Since AcEPs were shown to generate radicals, which have the potential to trigger lipid peroxidation, it was obvious to study the influence of AcEPs on mitochondrial coupling in LtP. In our experiments, the basal respiration of LtP was inhibited by the ATP synthase inhibitor Oligo, which blocks the proton channel of the ATP synthase. In addition, we applied compounds as potential uncouplers (Figure 7). CCCP, the prototype of an uncoupler, triggers a regain of LtP oxygen consumption after Oligo-induced inhibition of the cellular respiration by uncoupling the electron transfer from phosphorylation (Figure 7A). Of the AcEPs used in this experiment, AcEP1129 showed a comparable, yet smaller regain of the oxygen consumption (Figure 7F). This effect is visible at concentrations of 25 µM of AcEP1129 and declines at higher concentrations (which is similar to CCCP). The data prove at least for AcEP1129 that it uncouples leishmanial mitochondria at concentrations relevant for parasite killing.

Possible strategies to mitigate macrophage toxicity for AcEPs could involve introduction of larger substituents, which prevent premature activation and increase binding to heme, as well as combination of AcEPs with mitochondrial targeting residues as leishmanial mitochondria are essential for parasite survival. On the pharmacokinetic level, encapsulation of AcEPs in anionic liposomes or for cutaneous leishmaniasis local activation of AcEPs by photodynamic approaches are possible strategies to achieve more selective pharmacological activities.

## 5. Conclusions

In summary, certain AcEPs were shown (i) to kill *Leishmania* efficiently in vitro, (ii) to quickly react with Fe^2+^ under formation of oxygen- and carbon-centered radicals, (iii) to produce carbon-centered radicals in *Leishmania* which secondarily trigger CMH oxidation (superoxide radical formation) in LtP, and (iv) to impair mitochondrial functions in LtP during parasite killing. Although not all effects were seen with all AcEPs, this could have experimental reasons and does not exclude a contribution of these effects to the action of all AcEPs.

The data of different AcEPs demonstrate that their structures besides the peroxo bridge strongly influence their activity and mechanism of their antileishmanial action. Future studies should focus on overcoming host cell toxicity of AcEPs.

## Figures and Tables

**Table 1:** IC_50_ values of AcEPs along with Pen, Ac, AQ. Values were obtained from resazurin, MTT, or MTS viability assays using serial dilutions of the compounds with an endpoint at 48 h using 2 × 10^6^ LtP/mL, 6 × 10^6^ axLtA/mL, 5 × 10^5^ LdP/mL, or 10^5^ J774/mL, respectively. Data represent mean ± SD from at least 3–8 experiments.

Compound	IC_50_ (µM) Mean ± SD			
	LtP	axLtA	LdP	J774
Pen	0.84 ± 0.43	0.67 ± 0.31	0.55 ± 0.11	14.37 ± 6.48
Ac	>400	>400	n.d.	>400
AQ	103.32 ± 17.82	369.03 ± 9.81	n.d.	116.69 ± 24.29
AcEP1117	5.72 ± 1.92	56.40 ± 14.97	2.65 ± 0.34	43.80 ± 36.12
AcEP1118	1.00 ± 0.73	6.43 ± 0.40	4.10 ± 0.94	4.34 ± 1.91
AcEP1129	0.61 ± 0.21	3.29 ± 0.13	4.21 ± 0.36	0.90 ± 0.28
AcEP1130	0.22 ± 0.09	15.29 ± 0.31	39.48 ± 3.48	7.82 ± 0.47

**Table 2:** Reaction of AcEPs and other EPs with Fe^2+^. The compounds (50 µM) were incubated with 250 µM FeSO_4_ in the presence of 125 µM xylenol orange (XO) in a mixture of methanol/H_2_O (9:1 *v*/*v*). The formation of the XO/Fe^3+^ complex was monitored by measuring the OD at 560 nm vs. 650 nm over time. Data represent mean ± SD (*n* = 3).

Compound	Slope (nM·min^−1^) Mean ± SD
Asc	608 ± 52
Art	141 ± 8
AcEP1117	77 ± 18
AcEP1118	2940 ± 11
AcEP1129	200,213 ± 60,600
AcEP1130	146 ± 27

## Abbreviations

AA, antimycin A; Ac, anthracene; AcEP, anthracene endoperoxide; AQ, anthraquinone; Art, artemisinin; Asc, ascaridole; ATP, adenosine-5’-triphosphate; axLtA, axenic *Leishmania tarentolae* amastigotes; BHI, brain heart infusion; CCCP, carbonyl cyanide m-chlorophenylhydrazone; CMH, hydroxyl-3-methoxycarbonyl-2,2,5,5-tetramethylpyrrolidine hydrochloride; CM^•^, nitroxyl radical of CMH; DFO, deferoxamine; DMEM, Dulbecco´s Modified Eagle Medium; DMPO, 5,5-dimethyl-1-pyrroline N-oxide; DMSO, dimethyl sulfoxide; DTPA, diethylenetriaminepentaacetic acid; EP, endoperoxide; EPR, electron paramagnetic resonance; FBS, fetal bovine serum; FCS, fetal calf serum; Glu, glucose; IC_50_, half maximal inhibitory concentration; Ipp, peak to peak intensity; J774, J774 macrophage cell line; LtP, *Leishmania tarentolae* promastigotes; LdP, *Leishmania donovani* promastigotes; MTS, 3-(4,5-dimethylthiazol–2-yl)-5-(3-carboxymethoxyphenyl)-2-(4-sulphophenyl)-2H tetrazolium; MTT, thiazolyl blue tetrazolium bromide; ^1^O_2_, singlet oxygen; O_2_^•−^, superoxide radical; OD, optical density; Oligo, oligomycin; PBS, phosphate-buffered saline; Pen, pentamidine; PMS, phenazine methosulfate; PTFE, polytetrafluoroethylene; RB, rose bengal; RFLP, restriction fragment length polymorphism; SD, standard deviation; TEMP, 2,2,6,6-tetramethylpiperidine; TEMPO, (2,2,6,6-tetramethylpiperidine-1-yl)oxyl; UV, ultraviolet; XO, xylenol orange; YEM, yeast extract medium; WHO, World Health Organization
